# Reduction of peripheral regulatory T cells in active rheumatoid arthritis patients with coronary artery disease

**DOI:** 10.1186/s12865-021-00466-0

**Published:** 2021-12-16

**Authors:** Yanyan Wang, Rui Su, Baochen Li, Qiaoling Guo, Fangyuan Hu, Xiaopu Yu, Mingxia Ma, Lizhi Wang, Chong Gao, Xiaofeng Li, Caihong Wang

**Affiliations:** 1 Department of Rheumatology, The Second Hospital of Shanxi Medical University, Shanxi 030000 Taiyuan, China; 2 Department of Cardiology, The Second Hospital of Shanxi Medical University, Taiyuan, Shanxi China; 3 Department of Cardiology, Shanxi Cardiovascular Hospital, Taiyuan, Shanxi China; 4 Department of Pathology, Brigham and Women’s Hospital, Harvard Medical School, Boston, MA USA

**Keywords:** Lymphocyte, Regulatory T cell, Th17/Treg, Rheumatoid arthritis, Coronary artery disease

## Abstract

**Objective:**

To identify lymphocyte and CD4 + T cell subset characteristics, particularly regulatory T cells (Tregs), in active rheumatoid arthritis (RA) patients with coronary artery disease (CAD).

**Methods:**

A total of 54 RA patients with CAD (RA-CAD group), 43 RA patients without CAD (pure RA group), and 43 healthy controls (HC group) were enrolled. The absolute number and frequency of lymphocyte subpopulations and CD4 + T cell subsets were analyzed by flow cytometry. Serum levels of cytokines were analyzed using a cytometric bead array. Clinical and laboratory data were collected retrospectively and their correlation with CD4 + T subsets were analyzed.

**Results:**

There was a significant decrease in the absolute number of Treg cells (CD4 + CD25 + Foxp3 + T cells) in the RA-CAD group compared to the pure RA group (*p* < 0.001). Similarly, both the absolute number (*p* = 0.001) and frequency (*p* = 0.011) of Tregs in the RA-CAD group were decreased compared to the HCs, causing a Th17/Treg imbalance (*p* = 0.044). No difference was found in the absolute number and frequency of Treg cells between the pure RA and HC groups. However, the absolute Th17 cell count was increased in the pure RA group (*p* = 0.032). The serum level of cytokine IL-17 was lower in the RA-CAD group than in the pure RA group (*p* = 0.023). In the RA-CAD group, the Treg number was negatively correlated with the RA disease activity score and ESR value, and LDL and ApoB100 levels were negatively correlated with the number of Th17 cells.

**Conclusions:**

Active RA patients with CAD sustain more severe immune tolerance damage and Th17/Treg disorder. Monitoring of lymphocyte and CD4 + T cell subsets, particularly Treg cells, is crucial to understanding immune status in this group. Focusing on RA activity and CAD risk control, immune-regulatory therapy based on the Treg level may be more beneficial for RA patients with CAD.

**Supplementary Information:**

The online version contains supplementary material available at 10.1186/s12865-021-00466-0.

## Introduction

Rheumatoid arthritis (RA) is a common immune-mediated inflammatory disease characterized by chronic synovitis and vasculitis. Based on the pathological inflammatory mechanism, RA patients suffer from a wide range of other organ system injuries including heart, lung, and kidney, as well as polyarthritis. RA patients have a 48% higher morbidity and 50% higher mortality from cardiovascular disease (CVD) compared to the general population [1, 2], and approximately 50% of RA patients develop atherosclerosis, after excluding smoking, hypertension, hyperlipidemia, and other traditional risk factors [3]. RA is an independent risk factor for CVD.

In particular, coronary artery disease (CAD) occurs more frequently as a manifestation of CVD in RA, and RA patients have higher levels of all types of coronary plaque, contributing to future adverse cardiac events [4].This can be explained by the accumulation of classic risk factors for CAD, linked to the metabolic syndrome, and the systemic inflammatory load in RA [5]. Autoimmune disorders in RA patients induce activation of immune cells and the release of large amounts of inflammatory factors, which directly lead to vascular endothelial damage and platelet aggregation, accelerating the progression of atherosclerosis.

The immune-related molecular mechanisms that underlie the pathogenesis of CAD and RA have many similarities [6]. The consensus among experts is that immune dysfunction caused by multiple immune cell derangement is the root of RA, particularly the imbalance of Th17 and regulatory T (Treg) cells in peripheral blood [7–9]. Pro-inflammatory Th17 cells contribute to the induction and propagation of inflammation. As a subset of CD4 + T lymphocytes, Treg cells inhibit over-activated effector T cells and play an important role in the maintenance of autoimmune tolerance. Similarly, atherosclerotic lesions contain immunocytes from the blood. The components of cholesterol-carrying low-density lipoprotein trigger T cell proliferation and antibody production during the early stages of disease, and Treg cells negatively regulate this process [10, 11]. There is also a Th17/Treg imbalance in CAD patients [12]. However, few studies have reported Treg cell levels in RA patients with CAD.

Based on analyses of the clinical factors correlated with combined RA-CAD disease, which is different from pure RA disease, this study explored lymphocyte subsets, particularly Treg cell characteristics, revealing the immune status of this group and thereby providing guidance on immune-based treatment.

## Materials and methods

### Study design and participants

A total of 54 patients with a confirmed diagnosis of RA and CAD (RA-CAD group), and 43 pure RA patients without CAD or other heart disease (pure RA group) were selected from the Rheumatology Department, Second Hospital of Shanxi Medical University from January 2016 to January 2020; 43 healthy individuals were selected from the medical center as healthy controls (HC group). There were no statistical differences in age and sex among the three groups. All RA patients met the 2010 American College of Rheumatology/European League Against Rheumatism (ACR/EULAR) classification standard for RA [13] and had moderate-high disease activity scores (DAS28 > 3.2). The diagnosis of CAD was based on the 2013 European Society of Cardiology guideline [14] (23 patients after coronary angioplasty). Patients with other autoimmune diseases, severe cardiovascular disease such as NYHA class IV heart failure, tumors, infections, or other severe organ damage were excluded. Exclusions for the pure RA group were symptoms and signs of angina, electrocardiograph and/or ultrasound echocardiography showing myocardial ischemia, hypertension, diabetes and other indications of metabolic syndrome. Exclusions for the healthy control group were autoimmune diseases, CAD, and metabolic syndrome. Our study was approved by the Medical Ethics Committee of Shanxi Medical University (Approval (2020) YX No. 124), and all subjects provided written informed consent.

### Data collection

Clinical and laboratory parameters were collected retrospectively from the clinical database. The medical history included age, sex, BMI, clinical symptoms, and medications. Laboratory examinations included routine full blood count, blood lipid series, ESR, CRP, immunoglobulin (Ig), autoantibodies (including rheumatoid factor antigen [RF] and anti-cyclic citrullinated peptides [CCPs]), absolute number and frequency of peripheral blood lymphocyte subsets, and cytokine levels.

### Detection of lymphocytes by flow cytometry

All blood samples were collected on the morning of the patients’ consultation and tested immediately. All antibodies used for flow cytometry were purchased from BD Biosciences, Franklin Lakes, NJ, USA.

#### Detection of peripheral lymphocyte subsets

First, 50 µL mixed anticoagulant venous blood was added to two Trucount tubes labeled A and B using a reverse sampling method. Then, anti-CD3 (clone:SK7)/CD45 (clone:2D1)/CD4 (clone:SK3)/CD8 (clone:SK1) antibodies were added into tube A, and anti-CD3 (clone:SK7)/CD45 (clone:2D1)/CD16 (clone:B73.1)/CD56 (clone:NCAM16.2)/CD19 (clone:SJ25C1) antibodies were added into tube B. The contents of the tubes were kept at room temperature for 20 min, followed by incubation with 450 μL 1 × FACS hemolysin for 15 min and phosphate buffer saline (PBS) washing. Data were assessed via modified microbead-based single-platform flow cytometry (BD FACSCalibur, BD Biosciences), and a total of 15,000 cells were obtained by BD MultiSET software for testing (Additional file 1: Figure S1).

#### Detection of CD4 + T cell subsets

We followed the methods of a previous study [15]. For Th1/Th2/Th17 cell labeling, 10 µL 30 ng/mL phorbol 12-myristate 13-acetate, 10 μL 750 ng/mL ionmycin, and 1 μL GolgiStop (BD Biosciences) were added to 80 μL anticoagulated blood. This was cultured at 37 °C in a CO_2_ incubator for 5 h, cells were divided into tubes A and B, and anti-CD4 (clone:RPA-T4) was added for 20 min. Then the tubes were mixed with 1 mL fresh fixation/permeabilization liquid (BD Biosciences) and incubated for 30 min. Later, anti-IL-4 (clone:3010.211)/IFN-γ (clone:25,723.11) and anti-IL-17 (clone:SCPL1362) were added to tubes A and B, respectively, and they were incubated for 30 min in the dark. The samples were analyzed by flow cytometry (Additional file 1: Figure S2).

For Treg cell labeling, anti-CD4 (clone:RPA-T4)/CD25 (clone:M-A251) was added to 80 μL anticoagulated blood and incubated for 20 min in the dark. Then the tube was mixed with 1 mL fresh fixation/permeabilization liquid (BD Biosciences) and incubated for 30 min in the dark. Later, the cells were stained with anti-FOXP3 (clone:259D/C7) for 30 min and analyzed by flow cytometry after PBS washing within 24 h. A total of 10,000 cells in the portal were obtained and analyzed by BD CellQuest software (Additional file 1: Figure S2). The absolute number of each CD4 + T cell subset = percentage of positive cells in each subgroup × CD4 + T cell number (cells/μL).

### Detection of cytokines using a cytometric bead array (CBA)

After collecting venous blood, the serum was isolated and stored at − 80 °C to be tested. The serum levels of cytokines IL-2, IL-4, IL-6, IL-10, IL-17, TNF-α, and IFN-γ were analyzed using a magnetic bead-based multiplex immunoassay with a Human Th1/Th2/Th17 CBA kit (Jiangxi Cellgene Biotech Co., Ltd., Nanchang, Jiangxi, China) strictly following the manufacturer’s protocol. The BD FCAP Array software was used to acquire the data and the results are reported as pg/mL.

### Statistical analyses

The data were analyzed using SPSS V22.0 (IBM Corp., Armonk, NY, USA) statistical software. Results were expressed as mean ± SD or medians (Q25, Q75). Comparisons were performed using the independent samples t-test if the values followed a normal distribution; otherwise, the Mann–Whitney U test was used. The Kruskal–Wallis test (non-normality) was used to compare multiple group parameters. The enumeration data were expressed as percentages and were tested using the *χ*^2^ test. Spearman’s rank test was used for correlation analysis. *p* < 0.05 was considered statistically significant.

## Results

### General features of RA patients with CAD

The demographic characteristics and laboratory data of the participants are listed (Table 1). RA-CAD patients were mostly elderly and the proportion of males was 46%. We chose age- and sex-matched pure RA patients and HCs (*p* = 0.093; *p* = 0.909, respectively). Some patients in both groups had been on medical therapy, including glucocorticoids (prednisone 2.5–10 mg/day), DMARDS (MTX/LEF/HCQ), NSAIDS, and/or biological agents (rhTNFR-Fc), but there were no differences. Some RA-CAD patients received additional beta blockers, statins, antiplatelet agents, or other vasodilators (Table 2). There were no statistical differences in DAS28 scores, ESR, CRP, RF, and anti-CCP between the two groups, indicating that these patients had the same RA disease activity. The RA-CAD group had lower numbers of lymphocytes. For serum levels of lipoprotein, the low-density lipoprotein (LDL) values of the RA-CAD group were lower, but in the non-statin RA-CAD group there were no differences (data not listed). Serum levels of IgM and IgA were lower in the RA-CAD group than in the pure RA group; there were no significant differences in other assay indexes.

**Table 1:** Demographic and laboratory characteristics of the RA-CAD, pure RA, and HC groups

	RA-CAD (n = 54)	RA (n = 43)	HC (n = 43)	*p* value
Age (years)^a^	63.52 ± 9.35	60.74 ± 6.29	60.91 ± 3.63	0.093
Male/female^b^	25/29	19/24	18/25	0.909
BMI^a^	24.04 ± 3.56	22.75 ± 3.55	23.70 ± 3.69	0.222
Smoking: male/female^b^	18 (72.0%)/0	13 (68.4%)/0	9 (50.0%)/0	0.302
Disease duration (years)^c^	5.00 (0.50, 12.00)	5.00 (1.50, 11.00)	–	0.434
DAS28 score^c^	8.41 (5.93, 13.61)	8.90 (4.98, 15.46)	–	0.847
ESR (mm/h)^c^	52.50 (27.00, 84.00)	43.00 (25.00, 72.00)	–	0.459
CRP (mg/L)^c^	14.95 (5.54, 57.78)	21.80 (6.48, 41.70)	–	0.988
WBC (*10^9^/L)^d^	6.68 (5.34, 8.85)	6.90 (5.29, 7.50)	5.81 (5.12, 7.35)	0.152
LY (*10^9^/L)^d^	1.54 (1.16, 1.93)	1.66 (1.31, 2.14)	1.89 (1.61, 2.20)	0.009
MO (*10^9^/L)^d^	0.52 (0.39, 0.64)	0.53 (0.39, 0.59)	0.43 (0.34, 0.58)	0.303
PLT (*10^9^/L)^d^	257.50 (202.75, 293.25)	256.00 (215.00, 330.00)	230.00 (198.00, 274.00)	0.068
TC (mmol/L)^d^	3.71 (3.11, 4.27)	3.81 (3.23, 4.87)	4.24 (3.61, 4.69)	0.063
TG (mmol/L)^d^	1.17 (0.91, 1.66)	1.07 (0.76, 1.54)	1.12 (0.89, 1.57)	0.414
HDL (mmol/L)^d^	1.04 (0.91, 1.27)	1.18 (1.05, 1.28)	1.24 (1.01, 1.37)	0.061
LDL (mmol/L)^d^	1.93 (1.55, 2.38)	2.28 (1.88, 2.89)	2.36 (2.02, 2.87)	0.005
ApoA1 (g/L)^c^	1.13 (0.97, 1.34)	1.14 (1.02, 1.23)	–	0.813
ApoB100 (g/L)^c^	0.70 (0.57, 0.88)	0.78 (0.57, 1.01)	–	0.369
RF (IU/mL)^c^	40.00 (20.00, 160.00)	80.00 (20.00, 300.00)	–	0.225
Anti-CCP (IU/mL)^c^	433.65 (149.10, 1058.23)	829.80 (73.80, 1600.00)	–	0.218
IgG (g/L)^c^	12.30 (10.55, 15.85)	12.80 (8.92, 16.00)	–	0.912
IgM (g/L)^c^	2.32 (1.63, 3.56)	3.14 (2.20, 3.95)	–	0.037
IgA (g/L)^c^	1.01 (0.63, 1.33)	1.25 (0.87, 1.82)	–	0.007

ESR: Erythrocyte sedimentation rate; CRP: C-reactive protein; WBC: white blood cell; LY: lymphocyte; MO: monocyte; PLT: platelet; TC: total cholesterol; TG: triglyceride; HDL: high-density lipoprotein; LDL: low-density lipoprotein; ApoA1: apolipoprotein-A1; ApoB100: apolipoprotein-B100; RF: rheumatoid factor; Anti-CCP: antibodies to cyclic citrullinated peptides; Ig: immunoglobulin

^a^Data are mean ± SD and compared using the independent samples t-test

^b^Data are number (n)/percentage (%) and measured using the chi-square test

^c^Data are medians (Q25, Q75) and compared using the Mann–Whitney U test

^d^Data are medians (Q25, Q75) and compared using the Kruskal–Wallis test

**Table 2:** Clinical characteristics and treatments of the RA-CAD and pure RA groups

	RA-CAD (n = 54)	RA (n = 43)	*p* value
Morning stiffness > 1 h	32 (59.3%)	21 (48.8%)	NS
Dry mouth (%)	25 (46.3%)	19 (44.2%)	NS
Dry eye (%)	12 (22.2%)	11 (25.6%)	NS
Back pain (%)	8 (14.8%)	5 (11.6%)	NS
Treated/untreated	33/21	28/15	NS
GCs (prednisone 2.5–10mgday) (%)	19/54 (35.2%)	18/43 (41.9%)	NS
DMARDs (MTX/LEF/HCQ) (%)	23/54 (42.6%)	18/43 (41.9%)	NS
NSAIDs (%)	16/54 (29.6%)	15/43 (34.9%)	NS
Biologics (rhTNFR-Fc) (%)	5/54 (9.3%)	1/43 (2.3%)	NS
Statins (%)	32/54 (59.3%)	–	–
Anti-platelet drug (%)	25/54 (46.3%)	–	–
Beta-blockers (%)	9/54 (16.7%)	–	–
ACEI/ARBs (%)	4/54 (7.4%)	–	–
Coronary-expansion drugs (%)	10/54 (18.5%)	–	–

Data are number (n)/percentage (%) and were measured using the chi-square test

NS; Not significant; GCs: glucocorticoids; DMARDs: disease-modifying antirheumatic drugs; NSAIDs: nonsteroidal anti-inflammatory drugs; MTX: methotrexate; LEF: leflunomide; HCQ: Hydroxychloroquine; rhTNFR-Fc: recombinant human tumor necrosis factor-α receptor II: IgG Fc fusion protein for injection

### No differences Treg cells between the pure RA and HC groups

**Fig. 1:**
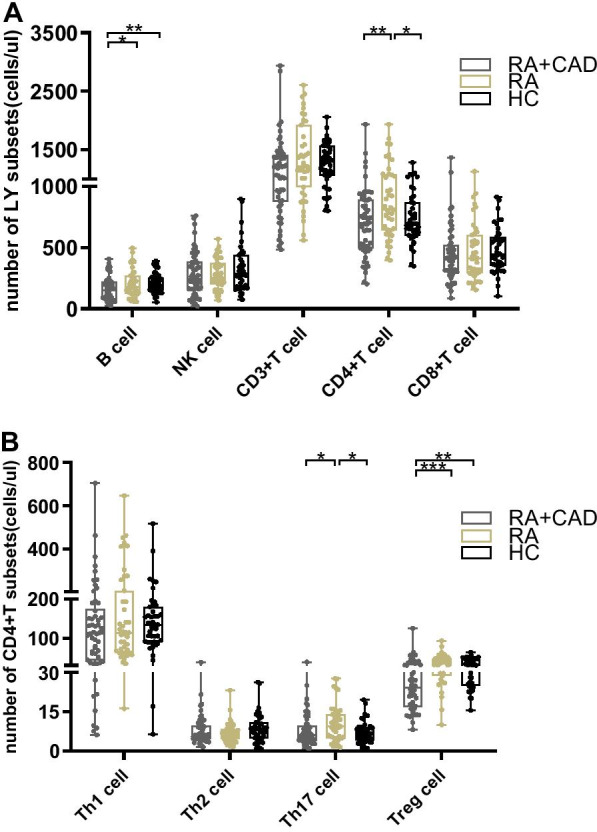
Comparison of lymphocyte and CD4 + T cell subset numbers among the RA-CAD (n = 54), pure RA (n = 43), and HC (n = 43) groups. **A** The number of B, NK, CD3 + T, CD4 + T, and CD8 + T cells in RA-CAD patients, pure RA patients, and healthy controls. **B** The number of Th1, Th2, Th17, and Treg cells in RA-CAD patients, pure RA patients, and healthy controls. Data are medians (Q25,Q75) and were compared using the Mann–Whitney U test between each of the two groups. *p < 0.05; **p < 0.01; ***p < 0.001

**Fig. 2:**
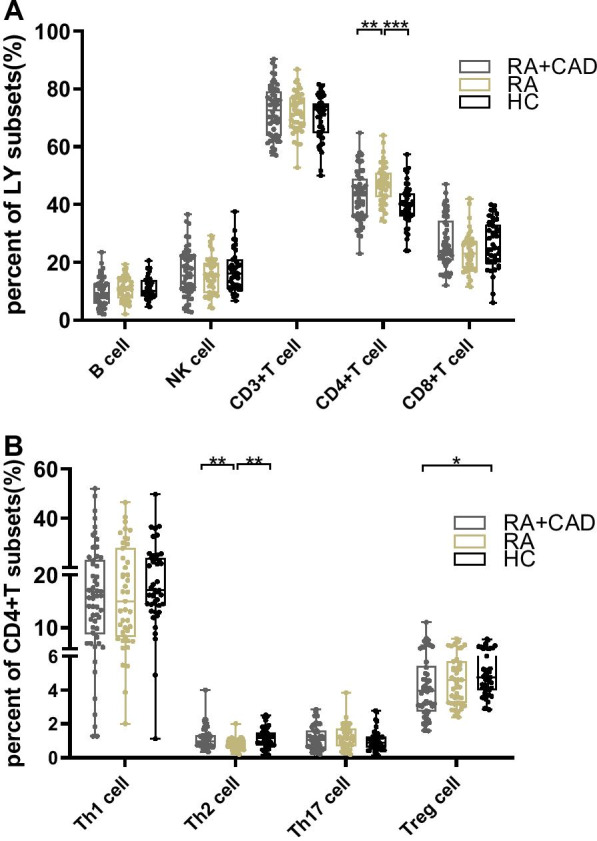
Comparison of lymphocyte and CD4 + T cell subset percentages among the RA-CAD (n = 54), pure RA (n = 43), and HC (n = 43) groups. **A** The percentages of B, NK, CD3 + T, CD4 + T, and CD8 + T cells in RA-CAD patients, pure RA patients, and healthy controls. **B** The percentages of Th1, Th2, Th17, and Treg cells in RA-CAD patients, pure RA patients, and healthy controls. Data are medians (Q25,Q75) and were compared using the Mann–Whitney U test between each of the two groups. *p < 0.05; **p < 0.01; ***p < 0.001

We found no differences in either absolute number or frequency (*p* = 0.235, *p* = 0.344, respectively) of Treg cells between the pure RA and HC groups. However, the absolute value of Th17 was increased in the pure RA group (*p* = 0.032) (Fig. 1B). The number and percentage of CD4 + T cells were higher in the pure RA group than in HCs (*p* = 0.035, *p* < 0.001, respectively) (Figs. 1A, 2A). The percentage of Th2 cells in the pure RA group was lower (*p* = 0.001) (Fig. 2B). There were no differences in B, NK, CD3 + T, CD8 + T, or CD4 + IFN-γ + Th1 subsets between the two groups (Additional file 1: Table S1, S2).

### Peripheral Treg cells are significantly reduced in the active RA-CAD group compared to the HC group

**Fig. 3:**
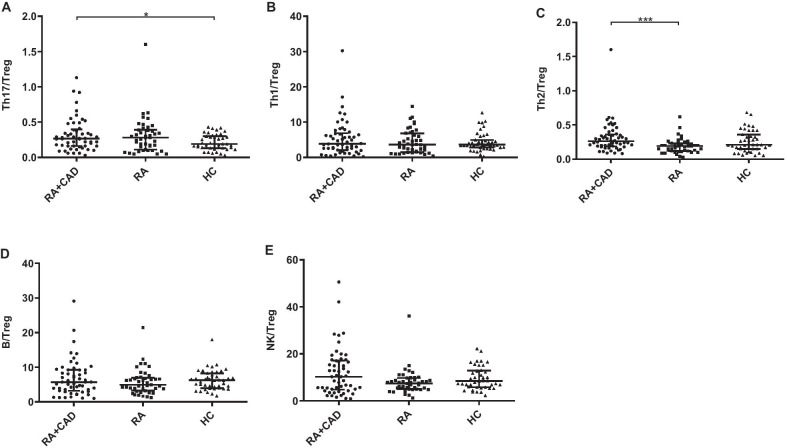
The ratio of Th17, Th1, Th2, B, and NK among the RA-CAD (n = 54), pure RA (n = 43), and HC (n = 43) groups. **A** The ratio of Th17/Treg. **B** The ratio of Th1/Treg. **C** The ratio of Th2/Treg. **D** The ratio of B/Treg. **E** The ratio of NK/Treg. Data are medians (Q25,Q75) and were compared using the Mann–Whitney U test between each of the two groups. *p < 0.05

The effects of lymphocytes on both RA and CAD have been described. However, few studies have investigated lymphocyte subset characteristics in RA patients with CAD. We compared the lymphocyte subsets between active RA-CAD patients and HCs. The absolute number of Treg cells was significantly lower in the RA-CAD group (*p* = 0.001) as well as Treg frequency (*p* = 0.011) (Figs. 1B, 2B). For Th17 cells, there was no significant difference. Decreased Treg cells led to a Th17/Treg imbalance (elevated ratio) in the RA-CAD group (*p* = 0.044) (Fig. 3A), while Th1/Treg (Fig. 3B), Th2/Treg (Fig. 3C), B/Treg (Fig. 3D) and NK/Treg (Fig. 3E) were the same as HC group. The B cell number was also lower (*p* = 0.009) (Fig. 1A). We found no differences in the NK, CD3 + T, CD4 + T, CD8 + T, CD4 + IFN-γ + Th1, or CD4 + IL-4 + Th2 subsets (Additional file 1: Table S1, S2).

### Significantly lower absolute number of peripheral Treg cells in the active RA-CAD group than in the pure RA group

To investigate whether lymphocyte changes were associated with CAD in RA patients, we compared the absolute number of peripheral lymphocyte subsets between RA-CAD patients and pure RA patients. The number was much lower in the RA-CAD group (*p* < 0.001) together with reduced Th17 cells (*p* = 0.032) (Fig. 1B). Total CD4 + T and B cell numbers were also reduced in the RA-CAD group (*p* = 0.043, *p* = 0.008, respectively) (Fig. 1A). For the other frequency analyses, the RA-CAD group had fewer CD4 + T cells (*p* = 0.049) and more Th2 cells (*p* = 0.024) compared to the pure RA group (Fig. 2A, 2B). There were no differences in the NK, CD3 + T, CD8 + T, or CD4 + IFN-γ + Th1 subsets between the two groups (Additional file 1: Table S1, S2).

### No difference in Treg cells between untreated and treated RA-CAD patients

Patients in the RA-CAD group were divided into untreated (n = 21) and treated (n = 33) groups (untreated was defined as never having received any treatment or only intermittent use of painkillers or Chinese herbal medicine), but no differences in Treg number or frequency were found (*p* = 0.950, *p* = 0.986, respectively); the same was true for other cell populations (Additional file 1: Table S3). In addition, due to the tendency for RA-CAD patients to have metabolic syndrome, with high blood pressure (HBP), diabetes (T2DM), and cerebral infarction, we regrouped RA-CAD into RA-CAD with T2DM and RA-CAD without T2DM groups, and RA-CAD with HBP and RA-CAD without HBP groups. Lymphocyte numbers between these subgroups showed no obvious differences, which eliminated the influence of confounding factors (data not listed).

### Lower serum levels of cytokine IL-17 in the RA-CAD group than in the pure RA group

**Fig. 4:**
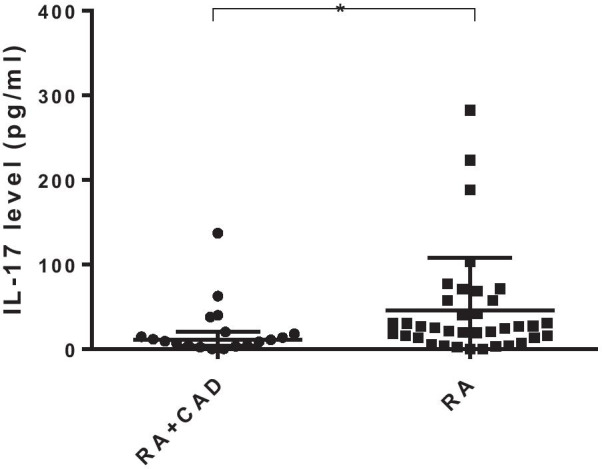
IL-17 level in the RA-CAD (n = 19), and pure RA (n = 37) groups. Data are medians (Q25,Q75) and were compared using the Mann–Whitney U test. *p < 0.05

Serum levels of cytokine IL-2, IL-4, IL-6, IL-10, IL-17, IFN-γ, and TNF-α were analyzed in 19 RA-CAD and 37 pure RA patients. The level of IL-17 was lower in the RA-CAD group (*p* = 0.023) (Fig. 4), consistent with a decline in Th17 cells, while other cytokines did not show statistically significant differences (Additional file 1: Table S4).

### Correlation analysis of Treg and Th17 cells with related clinical indicators in the RA-CAD group

**Fig. 5:**
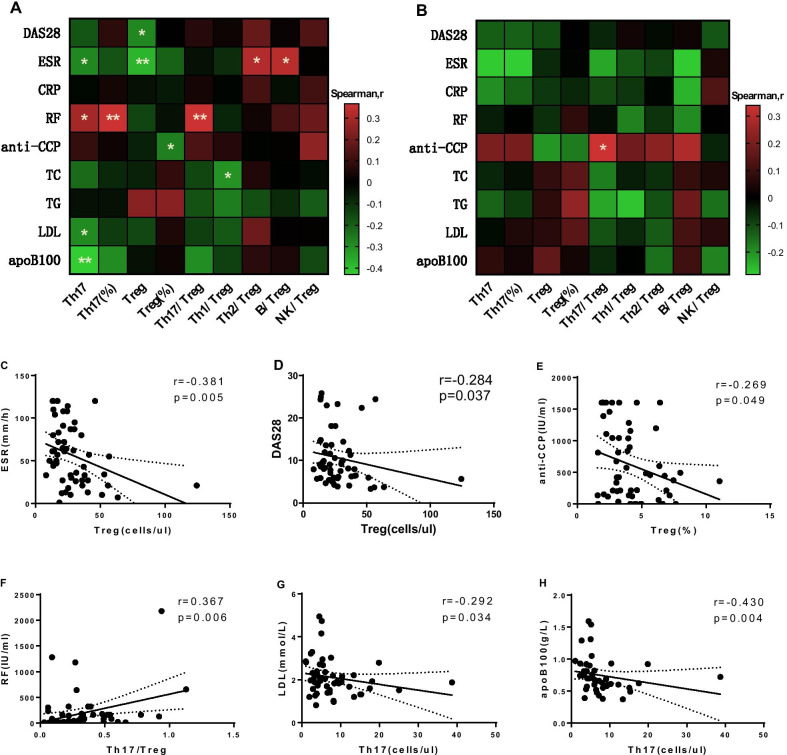
Correlation of Treg, Th17, and CD4 + T cell subset ratios with related clinical indicators in the RA-CAD (n = 54), and pure RA (n = 43) groups. **A** Heatmap of correlation of Treg, Th17, and CD4 + T cell subset ratios and related clinical indicators in RA-CAD patients. **B** Heatmap of correlation of Treg, Th17, and CD4 + T cell subset ratios and related clinical indicators in pure RA patients. **C**–**H** Correlation between Treg, Th17, and typical clinical indicators in RA-CAD patients. Correlations were assessed using Spearman’s rank test. *p < 0.05, **p < 0.01

We explored the correlation between Treg and Th17 cells and related clinical indexes. Treg number was negatively correlated with DAS28 score and ESR (r = −0.284, *p* = 0.037; r = −0.381, *p* = 0.005, respectively) (Fig. 5A, C, D). And the correlation with ESR remained significant even when a sample with uniquely high Treg numbers was excluded (r = −0.358, *p* = 0.008) (Additional file 1: Figure S3A), while the correlation with DAS28 scores fell just short of statistical significance (r = −0.256, *p* = 0.064) (Additional file 1: Figure S3B). In addition, Treg frequency was negatively correlated with the anti-CCP level (r = −0.269, *p* = 0.049) (Fig. 5A, E), and Th17/Treg was positively correlated with the RF level (r = 0.367, *p* = 0.006) (Fig. 5A, 5F), all consistent with the characteristics of RA disease. LDL and ApoB100 are risk factors for CAD. Lipoproteins containing ApoB100 are ligands for the LDL receptor, and the serum contents varied uniformly (r = 0.846, *p* < 0.001). Furthermore, we found that LDL and ApoB100 were negatively correlated with the number of Th17 cells (r = −0.278, *p* = 0.042; r = −0.430, *p* = 0.004, respectively) (Fig. 5A, G, H). LDL and ApoB100 were negatively correlated with the number of Th17 cells (r = −0.278, *p* = 0.042; r = −0.430, *p* = 0.004, respectively). In pure RA group, Th17/Treg was positively correlated with the anti-CCP level (r = 0.340, *p* = 0.026) (Fig. 5B).

## Discussion

This study included active RA patients with CAD hospitalized in the rheumatology department, and explored the immune status of this group by evaluating the levels of lymphocytes, particularly Tregs, and analyzing clinical indicators related to the disease. We showed that RA-CAD patients have lower CD4 + CD25 + Foxp3 + Treg cells than pure RA patients and HCs, causing a Th17/Treg imbalance; other lymphocytes and cytokines also varied. In addition, the number of Th17 and Treg cells were related to certain inflammatory factors and lipoprotein, showing that immunocytes, particularly Th17/Treg cells, play an important role in RA-CAD disease.

Various immune cells are involved in both RA and CAD. With deeper understanding of the immune environment in RA, the classic Th1/Th2 imbalance paradigm gradually turned to a Th17/Treg imbalance paradigm [16]. Some experts have already reported increased numbers of Th17 cells and Th1/Th17-related cytokines in the peripheral blood of RA patients; however, a lack of change, increases, and decreases in Treg cells have all been reported, and they vary during different stages of the disease [8, 9, 17]. In our research, we found higher numbers of Th17 cells in pure RA patients but no differences in Treg cells, indicating that a lymphocyte disorder exists during the course of the disease. In RA, inflammation and atherosclerosis are closely linked. Abnormal immune responses have been identified in atherosclerosis patients [18]. A high concentration of LDL-cholesterol is oxidized to oxLDL. Macrophages take up lipoproteins and transform into foam cells, gradually forming a necrotic core, while the ‘shoulder’ regions are full of activated T cells [11, 19]. Other innate immune cells are also minor participants [20]. There are a few reports on the characteristics of lymphocyte subsets in RA-CAD patients. Winchester [21] reported significant increases in circulating CD4 + T cell subsets in RA patients with subclinical coronary atherosclerosis. By contrast, in our study, the number of CD4 + T cells was reduced in the RA-CAD group. A previous study reported a vital role of an altered redox state for T cell function in RA patients [22]. Another study indicated that the oxidative inactivation of T-cell receptor-related enzymes causes decreased responsiveness of circulatory CD4 + T cells [23]. CD4 + Th cells assist B-cell function. This may explain the limited CD4 + T and B cells in RA-CAD patients with a higher oxidative stress level compared to the pure RA and HC groups. We also found an increased Th2 cell percentage in the RA-CAD group compared to the pure RA group. The main Th2 cytokine is IL-4, which suggests a disease‑promoting effect, while Th2-secreted IL-5 and IL-13 have a protective effect in atherosclerosis patients [10]. Our results hint at a proinflammatory role of Th2 cells in RA-CAD patients.

In our study, the key finding was the reduced numbers of CD4 + CD25 + Foxp3 + Treg cells in the RA-CAD group compared to both the pure RA and HC groups, causing a Th17/Treg imbalance and suggesting that Treg cells may play a crucial role in RA-CAD disease. Treg cells are a class of negative regulatory T cells expressing the IL-2 receptor α-chain (CD25) and intracellular transcription factor Foxp3, which are necessary to maintain immune homeostasis. Peripheral CD4 + T cells are induced by TGF-β to upregulate Foxp3 expression and they differentiate into inducible Tregs (iTreg) after antigenic stimulation, while natural Tregs (nTreg) mature directly in the thymus and are immunosuppressive [24]. Studies of Tregs build our understanding of immune control mechanisms and immune-mediated disease.

RA occurs due to failure of the immune tolerance mechanism to prevent the amplification of auto-reactive T cells. Impaired T cell proliferation and excess cytokine production are pivotal in the pathogenesis of RA and CAD. It is clear that Tregs have a protective effect during the process of atherogenesis [10, 11, 19]. Depletion of CD4 + CD25 + Treg aggravates atherosclerosis in hypercholesterolemic mice, whereas the input of Tregs is protective [25, 26]. In one study, Treg cells were virtually absent in normal intima but varied during all stages of plaque development [27]. The impaired intima also contained relatively few Foxp3 + Tregs (0.5–5% of CD3 + T cells) compared to inflammatory skin lesions (approximately 25% of CD3 + T cells), suggesting that local tolerance protection was impaired. Treg cells inhibit the development of autoimmunity by controlling the activity of effector T cells such as Th1 and Th17. Th17 cells play a proinflammatory role in RA [8], but paradoxical data have been reported regarding Th17 in CAD. Some studies have suggested that Th17 cells promote plaque stability by enhancing collagen deposition, which leads to increased fibrous cap formation [11], while others have suggested that oxLDL activates Th17 to promote atherosclerotic progression through several pathways [28]. A few studies have found higher peripheral Th17 and lower Treg cells, and attributed atherosclerosis to a Th17/Treg imbalance [12, 29]. In our study, both Treg number and frequency significantly decreased in the RA-CAD group, but Th17 cells were not significantly different compared to the HC group, suggesting more severe immune tolerance disability with the double inflammation caused by RA and atherosclerosis. Our results indicate that Treg cells are more responsible for the imbalance of Th17/Treg and play an important role in RA-CAD disease.

Two autoantigens have emerged as potentially important in the promotion of atherosclerosis: heat-shock protein 60 and LDL [11]. In our study, LDL values of the RA-CAD group were lower, but in the non-statin RA-CAD group there were no differences, showing that statin use was the driving force in this trend. Both oxLDL and native LDL contain several T/B cell epitopes. Some CD4 + Th cells react to peptide motifs from the ApoB100‑protein of LDL, which are represented by antigen-presenting cells on major histocompatibility complex class II molecules [5, 11, 19]. Th17 cells are thought to stabilize plaque; the negative correlation between LDL, ApoB100 and Th17 numbers in the RA-CAD group in our study suggests that Th17 may play an inhibitory role in the course of atherosclerosis. This view was also supported by the decreased Th17 and IL-17 levels in the RA-CAD group compared to the pure RA group. Foxp3 + Treg inhibits atherosclerosis by modulating lipoprotein metabolism [26]. ApoB may bind to enolase-1 expressed on the surface of immune cells to aggravate arthritis in RA patients [30]. This suggests a novel mechanism by which lipid metabolism regulates chronic inflammation in RA. However, whether Th17 and Treg cells are antigen-specific for LDL in RA-CAD patients requires confirmation by further study.

The management of RA-CAD includes control of traditional risk factors, platelet inhibition, and myocardial protection. RA serves as an independent risk factor, and the control of disease activity has also been emphasized in the EULAR recommendations [31]. In the treatment of RA, glucocorticoids and NSAIDs are considered protective, while some DMARDs and biologicals are considered to increase cardiovascular risk [32]. However, in active RA-CAD patients, there is a significant decrease in absolute Treg numbers. Recently, our department proposed using immune regulation therapy and achieved considerable success with it [33–35]. Our research shows that low-dose IL-2 or rapamycin can stimulate immune cell proliferation, particularly anti-inflammatory Tregs. Treg-targeting therapy is also a novel approach for preventing atherosclerosis [36]. Immune-modulation may have a palliative effect for both RA and CAD disease activity.

This study had some limitations. First, it was a single-center study. In addition, because it was a retrospective study, some data were missing or undetectable. Statin therapy may ameliorate the Th17/Treg imbalance, and the impact of statins on Tregs cannot be ignored [29]. Further multi-center prospective studies are required to provide a basis for precision and personalized medicine.

## Conclusions

This study confirms that active RA patients with CAD have more extreme immune tolerance injury, and the primary concern for clinicians is the Th17/Treg imbalance. It is important to pay attention to decreased Treg cells while controlling the primary disease. Simply pursuing disease activity control and serologic remission while ignoring the long-term negative cardiovascular effects is inadvisable. The central issue is to find a balance between therapeutic efficiency and safety. Thus, clinicians should be alert to the absolute reduction of Treg cell numbers in RA-CAD patients during treatment, and choose optimal therapy on the basis of laboratory reports. In addition, the detailed mechanism underlying the decreased levels of Treg cells should be further explored.

## Supplementary Information

**Additional file 1. Table S1-S3**. The number and percentages of lymphocyte and CD4+T cell subset in different groups; **Table S4**. Cytokine levels in the RA-CAD and pure RA groups; **Figure S1-S2**. Phenotypic characterization of lymphocyte and CD4+T cell subsets via flow cytometry; **Figure S3**. Correlation between Treg number and disease activity indicators when a sample with uniquely high Treg number was excluded. 

## Data Availability

All data generated or analyzed during this study are included in this article.
