# Supplementary material for: Reduction of peripheral regulatory T cells in active rheumatoid arthritis patients with coronary artery disease
Source: BMC Immunol. 2021 Dec 16;22:76. doi: 10.1186/s12865-021-00466-0 (PMC8680032; doi:10.1186/s12865-021-00466-0)
Supplement: Supplementary file 1 — Additional file 1. Table S1-S3. The number and percentages of lymphocyte and CD4+T cell subset in different groups; Table S4. Cytokine levels in the RA-CAD and pure RA groups; Figure S1-S2. Phenotypic characterization of lymphocyte and CD4+T cell subsets via flow cytometry; Figure S3. Correlation between Treg number and disease activity indicators when a sample with uniquely high Treg number was excluded. [file 12865_2021_466_MOESM1_ESM.docx]

**Table S1. The number of lymphocytes and CD4+T cell subset (cells/μL) in the RA-CAD (A), pure RA (B), and healthy control (C) groups.**

| Cell count  (cells/μL) | RA-CAD (A) (n = 54) | RA (B) (n = 43) | HC (C) (n=43) | p-value  A vs. B | p-value  A vs. C | p-value  B vs. C |
| --- | --- | --- | --- | --- | --- | --- |
| CD3+T | 1213.08 (873.52, 1418.30) | 1219.14 (993.90, 1918.90) | 1304.00 (1061.31, 1569.85) | 0.125 | 0.075 | 0.880 |
| B | 149.60 (77.22, 223.70) | 177.24 (125.65, 270.29) | 197.81 (150.43, 258.19) | **0.043** | **0.009** | 0.531 |
| NK | 243.68 (148.71, 383.57) | 253.34 (196.42, 372.43) | 286.63 (179.70, 437.20) | 0.576 | 0.150 | 0.358 |
| CD4+T | 699.71 (492.00, 890.01) | 808.62 (641.35, 1088.31) | 694.03 (598.00, 869.36) | **0.008** | 0.499 | **0.035** |
| CD8+T | 416.50 (302.18, 522.59) | 414.50 (290.50, 603.07) | 443.71 (354.75, 587.58) | 0.794 | 0.196 | 0.310 |
| Th1 | 112.78 (43.30, 175.05) | 113.81 (63.15, 206.46) | 134.40 (90.09, 179.59) | 0.323 | 0.148 | 0.509 |
| Th2 | 5.69 (4.60, 9.81) | 6.65 (4.51, 8.42) | 8.19 (4.73, 10.88) | 0.836 | 0.287 | 0.110 |
| Th17 | 6.01 (3.81, 9.71) | 9.38 (4.84,13.95) | 6.67 (4.07, 9.22) | **0.032** | 0.719 | **0.034** |
| Treg | 24.17 (16.79, 36.06) | 37.48 (28.67, 47.10) | 32.65 (25.03, 46.79) | **<0.001** | **0.001** | 0.235 |
| Th1/Th2 | 14.81 (6.99, 27.48) | 19.00 (11.05, 32.96) | 16.94 (9.96, 31.53) | 0.091 | 0.345 | 0.400 |
| Th17/Treg | 0.27 (0.16, 0.40) | 0.28 (0.11, 0.39) | 0.19 (0.13, 0.30) | 0.766 | **0.044** | 0.109 |
| Th1/Treg | 3.89 (2.03, 6.85) | 3.60 (1.47, 6.82) | 3.63 (2.81, 4.95) | 0.616 | 0.785 | 0.720 |
| Th2/Treg | 0.26 (0.19, 0.36) | 0.19 (0.12, 0.23) | 0.21 (0.12, 0.36) | **<0.001** | 0.157 | 0.064 |
| B/Treg | 5.71 (3.18, 9.24) | 4.95 (3.24, 6.98) | 6.24 (3.98, 8.24) | 0.470 | 0.799 | 0.220 |
| NK/Treg | 10.28 (4.81, 17.15) | 7.46 (5.04, 9.79) | 8.47 (5.83, 12.87) | 0.082 | 0.695 | 0.068 |

Data are medians (Q25,Q75) and were compared using the Mann-Whitney U test. CD3+T: CD3+T cells; B: B cells; NK: natural killer T cells; CD4+T: CD4+ T cells; CD8+T: CD8+ T cells; Th1: T helper 1 cells; Th2: T helper 2 cells; Th17: T helper 17 cells; Treg: regulatory T cells.

**Table S2.** **The percentages of lymphocytes and CD4+T cell subset (%) in the RA-CAD (A), pure RA (B), and healthy control (C) groups.**

| Cell count  (%) | RA-CAD (A) (n = 54) | RA (B) (n = 43) | HC (C) (n = 43) | p-value  A vs. B | p-value  A vs. C | p-value  B vs. C |
| --- | --- | --- | --- | --- | --- | --- |
| CD3+T | 72.65 (63.81, 79.15) | 71.48 (66.74, 77.09) | 72.78 (64.73, 75.00) | 0.896 | 0.378 | 0.450 |
| B | 9.00 (5.79, 12.56) | 10.78 (7.52, 13.77) | 10.06 (8.28, 13.84) | 0.265 | 0.112 | 0.832 |
| NK | 15.54 (10.13, 22.05) | 15.51 (9.52, 19.75) | 16.00 (11.10, 21.00) | 0.611 | 0.663 | 0.414 |
| CD4+T | 43.00 (36.00, 48.97) | 47.00 (42.56, 51.02) | 39.92 (36.00, 43.82) | **0.007** | 0.131 | **<0.001** |
| CD8+T | 24.03 (20.76, 34.34) | 23.16 (17.94, 27.22) | 24.83 (20.05, 32.92) | 0.155 | 0.833 | 0.117 |
| Th1 | 15.84 (8.81, 23.16) | 15.00 (8.27, 27.80) | 17.10 (14.23, 23.93) | 0.867 | 0.152 | 0.218 |
| Th2 | 0.95 (0.70, 1.32) | 0.72 (0.57, 1.04) | 1.14 (0.73, 1.49) | **0.003** | 0.561 | **0.001** |
| Th17 | 1.04 (0.57, 1.60) | 1.14 (0.70, 1.70) | 0.88 (0.62, 1.24) | 0.692 | 0.437 | 0.192 |
| Treg | 3.98 (2.75, 5.42) | 4.60 (3.24, 5.68) | 4.75 (4.01, 6.01) | 0.116 | **0.011** | 0.344 |

Data are medians (Q25,Q75) and compared using the Mann-Whitney U test.

CD3+T: CD3+T cells; B: B cells; NK: natural killer T cells; CD4+T: CD4+ T cells; CD8+T: CD8+ T cells; Th1: T helper 1 cells; Th2: T helper 2 cells; Th17: T helper 17 cells; Treg: regulatory T cells.

**Table S3. The number and percentages of lymphocyte and CD4+T cell subset in untreated RA-CAD (A), and treated RA-CAD (B) groups.**

| Cell  cells/μL or % | Untreated  Group (A) (n = 21) | Treated  Group (B) (n = 33) | p-value  A vs. B |
| --- | --- | --- | --- |
| CD3+T | 1215.13 (719.41, 1457.37) | 1211.03 (938.00, 1376.57) | 0.613 |
| CD3+T (%) | 73.30 (62.72, 77.79) | 70.52 (63.97, 80.00) | 0.790 |
| B | 141.89 (84.31, 188.10) | 168.48 (67.50, 276.70) | 0.267 |
| B (%) | 8.00 (5.97, 12.05) | 10.81 (5.30, 13.67) | 0.529 |
| NK | 231.58 (159.14, 384.24) | 255.77 (93.17, 401.30) | 0.908 |
| NK (%) | 17.90 (10.57, 23.12) | 15.00 (9.49, 22.00) | 0.511 |
| CD4+T | 695.00 (487.62, 898.85) | 720.67 (491.00, 889.67) | 0.506 |
| CD4+T (%) | 42.00 (36.00, 50.70) | 44.00 (35.50, 46.77) | 0.683 |
| CD8+ | 413.44 (232.22, 643.86) | 428.85 (310.80, 499.03) | 0.651 |
| CD8+T (%) | 23.55 (20.90, 31.92) | 24.27 (20.26, 35.93) | 0.664 |
| Th1 | 95.78 (47.97, 211.16) | 113.82 (42.36, 153.99) | 0.908 |
| Th1 (%) | 12.50 (9.04, 23.69) | 16.10 (8.00, 22.73) | 0.986 |
| Th2 | 8.55 (5.06, 10.11) | 5.24 (4.42, 8.93) | 0.175 |
| Th2 (%) | 1.16 (0.69, 1.75) | 0.80 (0.71, 1.26) | 0.283 |
| Th17 | 6.02 (4.04, 9.30) | 5.99 (3.40, 10.88) | 0.958 |
| Th17 (%) | 1.04 (0.50, 1.97) | 1.04 (0.62, 1.40) | 0.986 |
| Treg | 24.86 (16.59, 33.50) | 23.85 (16.60, 39.18) | 0.950 |
| Treg (%) | 3.96 (3.04, 4.73) | 4.00 (2.44, 5.89) | 0.986 |

Data are medians (Q25,Q75) and were compared using the Mann-Whitney U test.

**Table S4.** **Cytokine levels (pg/mL) in the RA-CAD and pure RA groups.**

| Cytokines (pg/mL) | RA-CAD (n = 19) | RA (n = 37) | p-value |
| --- | --- | --- | --- |
| IL-2 | 5.50 (1.96, 12.82) | 6.82 (4.16, 14.17) | 0.064 |
| IL-4 | 4.93 (1.67, 9.41) | 6.41 (3.61, 15.13) | 0.078 |
| IL-6 | 23.69 (10.93, 73.08) | 43.64 (15.96, 89.90) | 0.598 |
| IL-10 | 7.76 (4.54, 10.50) | 7.87 (5.65, 18.89) | 0.183 |
| IL-17 | 10.81 (4.04, 20.25) | 25.01 (13.21, 57.49) | **0.023** |
| IFN-γ | 6.10 (3.27, 13.84) | 8.24 (5.65, 17.80) | 0.213 |
| TNF-α | 10.49 (2.50, 29.04) | 14.04 (5.60, 31.90) | 0.203 |

Data are medians (Q25,Q75) and were compared using the Mann-Whitney U test.

IL-2: interleukin-2; IL-4: interleukin-4; IL-6: interleukin-6; IL-10: interleukin-10; IL-17: interleukin-17; IFN-γ: interferon-γ; TNF-α: tumor necrosis factor-α.


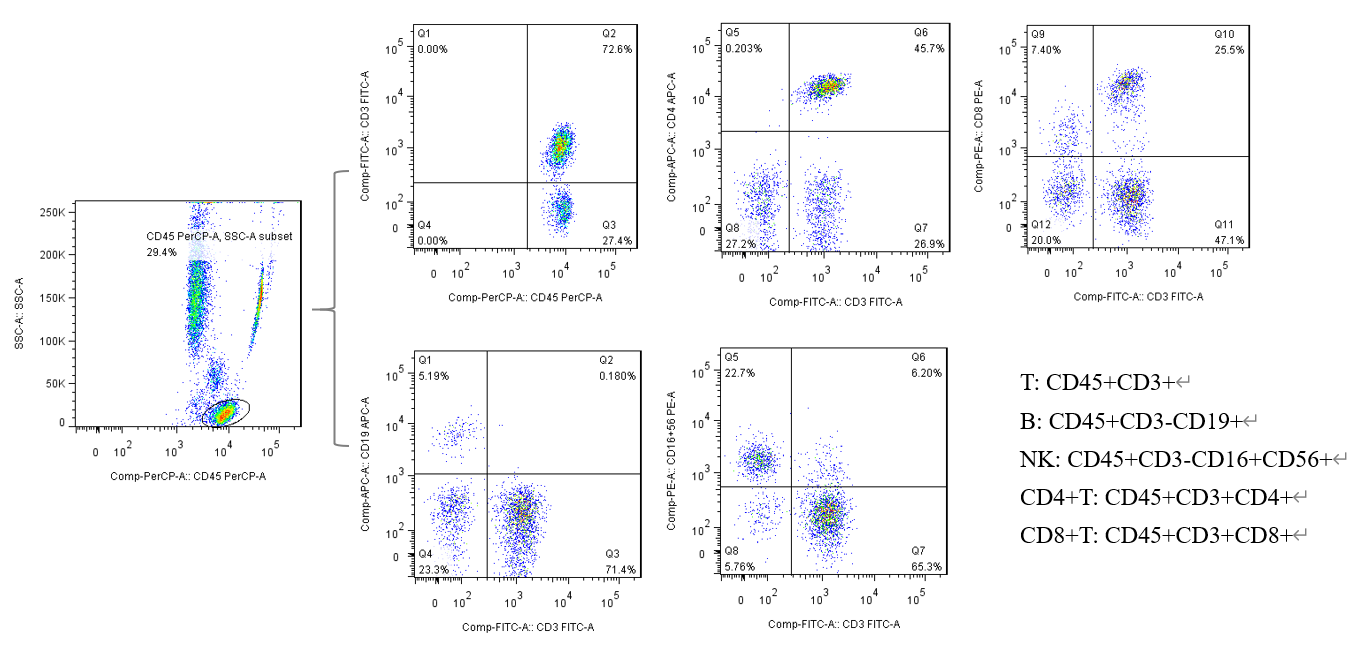


**Figure S1. Phenotypic characterization of lymphocyte subsets via flow cytometry.** T: CD45+CD3+; B: CD45+CD3-CD19+; NK: CD45+CD3-CD16+CD56+; CD4+T: CD45+CD3+CD4+; CD8+T: CD45+CD3+CD8+.

**
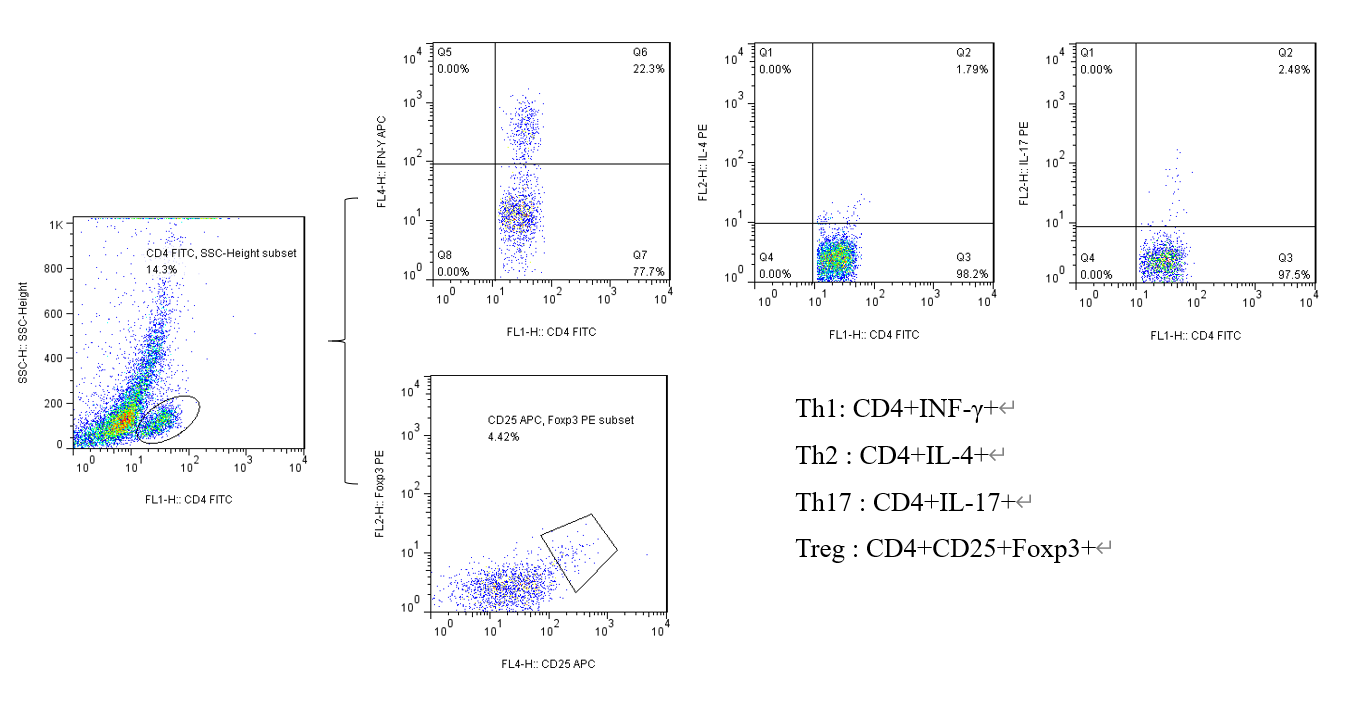
**

**Figure S2. Phenotypic characterization of CD4+T cell subsets via flow cytometry.**

All dot plot analysis is of CD4+ gated lymphocytes. Th1:CD4+INF-γ+; Th2: CD4+IL-4+; Th17: CD4+IL-17+; Treg: CD4+CD25+Foxp3+.

**Figure S3. Correlation between Treg number and disease activity indicators in RA-CAD (n = 54) group when a sample with uniquely high Treg number was excluded.**

**(A)** Correlation between Treg number and ESR**. (B)** Correlation between Treg number and DAS28 scores. Correlations were assessed using Spearman’s rank test.
